# Oceanic mantle reflections in deep seismic profiles offshore Sumatra are faults or fakes

**DOI:** 10.1038/s41598-019-49607-x

**Published:** 2019-09-16

**Authors:** Jean-Claude Sibuet, Enyuan He, Minghui Zhao, Xinming Pang, Frauke Klingelhoefer

**Affiliations:** 1 Key Laboratory of Ocean and Marginal Sea Geology, South China Sea Institute of Oceanology, Chinese Academy of Sciences, Guangzhou, 510301 China; 2 44 rue du Cloitre, 29280 Plouzané, France; 3 Ifremer Centre de Brest, B.P. 70, 29280 Plouzané, Cedex, France; 4 Innovation Academy of South China Sea Ecology and Environmental Engineering, Chinese Academy of Sciences, Guangzhou, 510301 China; 5 College of Earth and Planetary Sciences, University of Chinese Academy of Sciences, Beijing, 100049 China

**Keywords:** Geophysics, Seismology

## Abstract

In the late 90’s, some faults identified within oceanic crust were demonstrated to be artifacts arising from out-of-plane scattering along linear sediment-buried fault scarps. Symmetrical mantle reflections observed southwest northern Sumatra on seismic reflection profiles have been identified as faults cutting through the upper mantle down to unprecedented depths reaching ~45 km. Seawater being conveyed along sub-vertical re-activated fracture zones (FZs) to the upper mantle, the mantle portions of FZs are serpentinized and act as mirrors for seismic rays. We suggest that the mantle features are not faults but artifacts resulting from out-of-plane reflections on these mirrors. Two perpendicular seismic profiles crossing the same FZ display two dipping features down to 30 km, which cannot be explained as faults from recent tectonic and structural constraints but merely as out-of-plane reflections on this FZ. This result confirms that most of mantle reflections observed southwest northern Sumatra are fakes rather than faults.

## Introduction

With the fast development of multi-channel seismic (MCS) systems in the late 70’s, oceanic crustal features were interpreted as faults or magmatic layering in the middle and lower crust, without discussions concerning the possible existence of artifacts. The classical example concerns Ocean Drilling Program Hole 504B in the Panama basin, where a clear crustal dipping event was interpreted as a mechanical failure within the brittle upper oceanic crust^1^ and later as a low-angle fault striking perpendicular to the ridge axis^2^. From a specifically designed 1-km closely space mixed single-channel seismic (SCS) and MCS survey, Kent *et al*.^3^ demonstrated that this dipping event was a scattering artifact from an ~80-m high, E-W trending sediment-buried basement fault scarp located ~2 km south of Hole 504B. The interplay between the directions of the profile and of the basement fault scarp suggests that dipping events imaged in oceanic crust in some examples may be scattering artifacts rather than real geologic features.

Since the beginning of this century, seismic industry ships operating large seismic sources with up to 15-km long streamers have provided images not only of oceanic crustal reflections but also of numerous mantle reflections. Could we have the same interrogations concerning the interpretation of mantle reflections? Are all of them faults or some of them artifacts? A very good example to answer this question is the recently published MCS WG3 profile acquired in the oceanic domain seaward of the Sumatra trench displaying mantle reflections identified down to an unprecedented depth of 45 km^4^. Examples of MCS profiles acquired in similar geodynamic environments (Middle America trench^5–7^, Chile trench^8^, Aleutian trench^9^) show that these mantle reflections imaged within the upper 10 km and complemented by wide-angle reflection data are re-activated normal faults due to plate bending before subduction. These faults give rise to crustal and upper mantle tensional features. Seawater is carried down to the upper mantle, and low mantle velocities are interpreted to arise from a combination of fluid-filled fractures and mineral transformation by hydration, thus decreasing mantle densities and velocities^7^. The objective of this paper is not to discuss the interpretation of WG3 mantle features as faults^4,5,10^ or as a serpentinization front^11^ because these interpretations do not violate present-day geophysical/tectonic earth models, but to question the fact that some of these mantle features might be out-of-plane reflections on fracture zones (FZs) acting as vertical mirrors for seismic rays.

**Figure 1:**
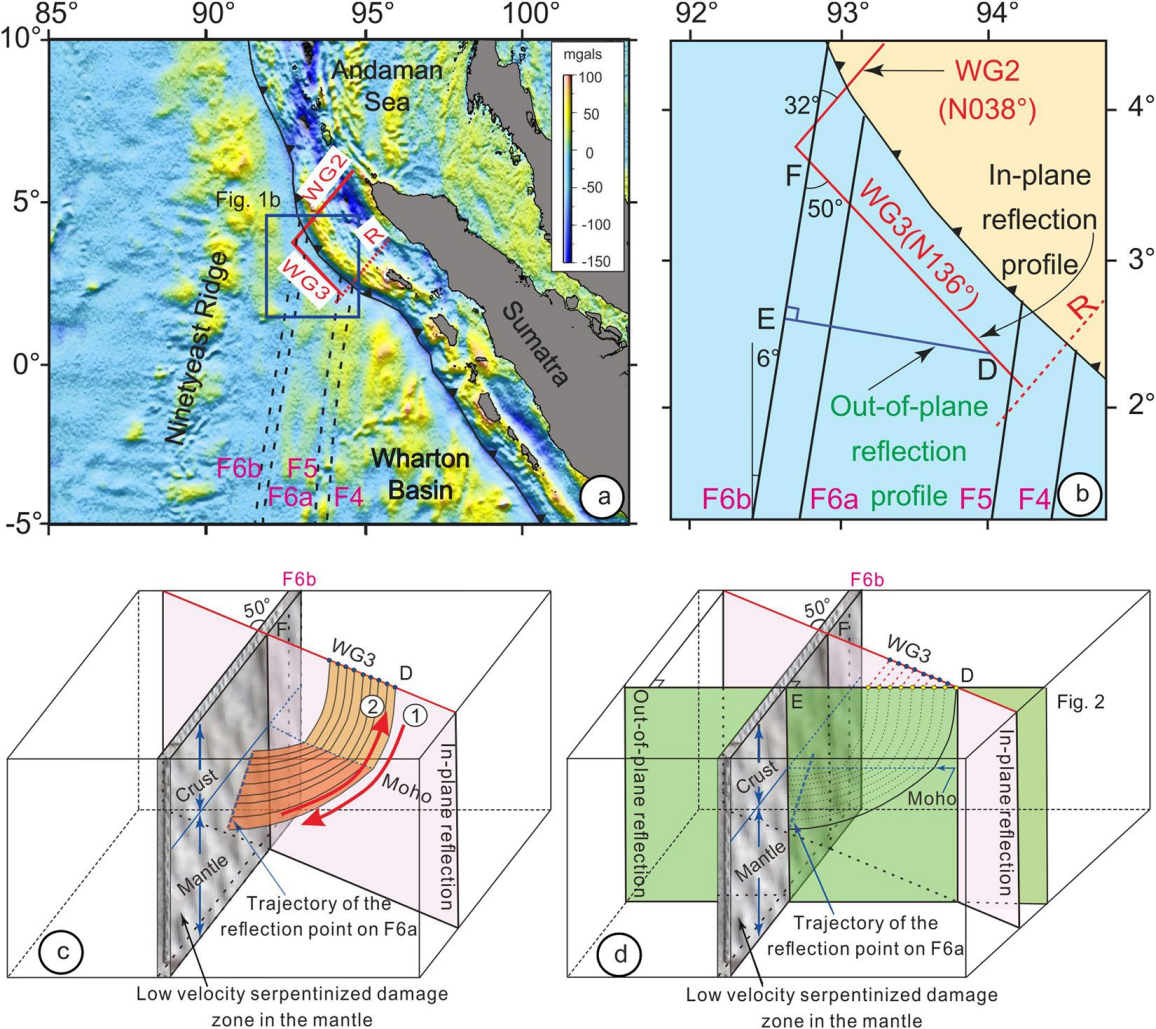
Location of seismic profiles WG3, WG2^4^ and refraction profile R^24^ on a gravity map computed from^14^). (**a**) Main FZs (F4, F5, F6a and F6b) are shown by dashed lines and profiles by red lines. (**b**) Close up with in-plane (DF) and out-of-plane (DE) reflection profiles. Black lines show main FZs. Oceanic domain, light blue; Sumatra accretionary wedge, light yellow. (**c**) 3D schematic view of the calculated ray paths of out-of-plane mantle reflections, which occur perpendicularly to F6b. 1 and 2 show an example of a two ways ray path. The Moho appears as a dark blue line. Ray paths in the water, sediments and oceanic crust above the Moho define a surface colored as the crust in Fig. 2 and ray paths in the mantle define a surface colored as the mantle in Fig. 2. (**d**) If the seismic ship is in D, out-of-plane reflections occur perpendicularly to the mantle portion of F6b. The ray path appears as continuous in this plane (colored in green). A few shots (blue dots) are shown along Profile WG3. For the fifth shot, the surface trace of the out-of-plane reflection (red line) appears backward of the green plane, showing the variations in the geometry of the recording system with the fixed in-plane reflections (light pink vertical plane), the fixed vertical plane of F6b (vertical plane with grey motifs), and the green out-of-plane reflections, which is moving in parallel to itself simultaneously with the ship moving along Profile WG3.

**Figure 2:**
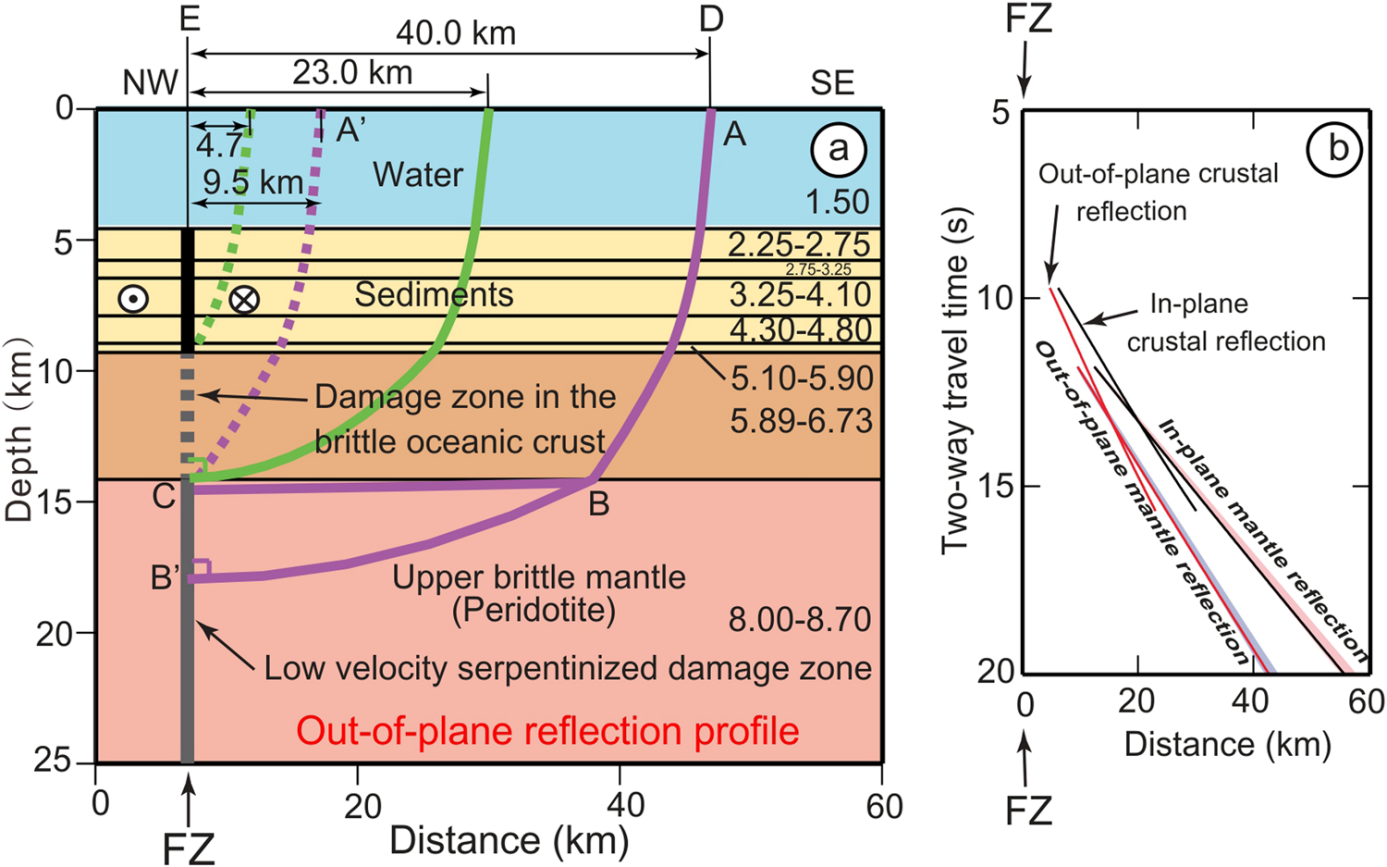
Out-of-plane reflection ray paths. (**a**) 2D velocity model based on the oceanic 1D oceanic model of Profile R located in Fig. 1^24^. Velocities are given in km/s. The vertical re-activated FZ consists of a deformed zone in the sediments (black solid line), a crustal damage zone (mylonite) (thick dashed dark grey line) and a mantle damage zone (low velocity serpentinized peridotite) (thick solid grey line). ABB’ is the computed ray path reflected perpendicularly to the sub-vertical FZ in the mantle. A’C is the ray path with the mantle ray path portion reduced to zero, the ship being 9.5 km away from the FZ. The dashed green line is the basement reflection. The continuous green line is the reflection on the crustal damage zone at the base of the crust, the ship being 23.0 km away from the FZ. (**b**) For the ABB’ ray path, the out-of-plane reflections have been computed by using the RayInvr software^23^. See text for computations of out-of-plane reflections projected along Profile WG3 (in-plane reflections). In (**b**), the light blue and light red areas represent travel time ranges corresponding to different mantle velocity distributions (See text for detailed explanations).

The 233 km-long WG3 seismic profile was acquired in the oceanic Wharton basin by WesternGeco operating a 12-km long streamer and a 10,000 in^3^ air gun source^4^. It is N136° oriented, nearly parallel to the Sumatra trench at a distance of 32 to 66 km, and oblique with respect to the N006° oriented left-lateral strike-slip FZs^12,13^ as identified on the gravity map computed from Sandwell *et al*.^14^ (Fig. 1a). Along Profile WG3, the oceanic crust is ~55–60 Ma old^15^ and numerous FZs were formed at that time and are re-activated^16^ since ~17.5 m.y^13^. In the overlying sediments, they are associated with flower structures and vertical offsets^4,10^ (Supplementary Fig. 1), suggesting strike-slip and normal faulting motions along them, the two sides of the FZ being sheared and pulled apart. Thus, seawater was probably transferred to the mantle through the sedimentary and crustal portions of FZs allowing subsequent serpentinization processes to occur in the mantle^4^ (Fig. 2a).

**Figure 3:**
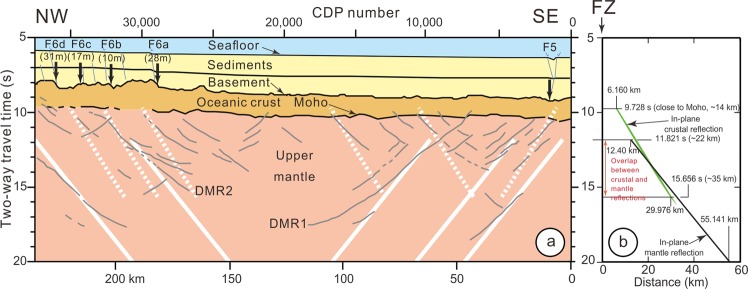
Interpretation of the WG3 seismic profile with identification of mantle features (thin light grey lines) interpreted as faults by Qin and Singh^4^. (**a**) Vertical arrows, location of FZs identified on the gravity map^15^ and by their post-spreading sedimentary deformations^10^. Out-of-plane mantle reflections are computed along Profile WG3 from 11.8 s to 20 s TWTT (solid white lines). They are coincident with five mantle features identified by Qin and Singh^4^. Out-of-plane crustal reflections are computed along Profile WG3 from 9.7 s to 15.7 s TWTT (dashed white lines). They are coincident with at least six mantle features identified by Qin and Singh^4^. (**b**) Computed in-plane crustal and mantle reflections along Profile WG3 (in-plane reflections) displayed at the same scale as in (**a)**. Note the overlap between out-of-plane crustal and mantle reflections between 11.8 and 15.7 s TWTT along WG3 Profile.

The top and the base of the oceanic crust are well imaged along Profile WG3 (Fig. 3a and Supplementary Fig. 1). Deep mantle reflections starting close to the Moho down to 45 km (Fig. 2a) are interpreted as deep penetrating mantle faults^4^. The number of mantle reflections roughly decreases with depth from 30 events in the upper mantle between the Moho (located at ~14 km) and 30–35 km to 7 events below 35 km^4^. This difference in number of events was proposed to be due to the presence of a serpentinized mantle layer overlying a pristine mantle layer located below 30–35 km down to the base of the lithosphere located at ~60 km, with a large stress drop occurring at the boundary between the two layers^4^.

However, these mantle features interpreted as faults (Fig. 3) raise numerous questions:

- Why does symmetry exist between mantle features trending N058° and −N058° in Fig. 3 and Supplementary Fig. 1b,c?
- Why can no systematic offsets be observed at the intersections of mantle features of opposite dips?
- Why do mantle features reach up close to the Moho interface with almost no extension into the oceanic crust and why are they disconnected from oblique crustal features?
- Why does the polarity of seismic signal change for the Deepest Mantle Reflector 1 (DMR1)?

Qin and Singh^4^ proposed explanations for some of the above observations discussed in detail in the following sections but not for all of them. We suggest that most of these mantle features are not faults but out-of-plane reflections which occur perpendicularly to sub-vertical FZs acting as vertical mirrors for seismic rays, a case study not explored by Carton *et al*.^10^ and Qin and Singh^4^ for example.

## Results

### Serpentinization process in the mantle portion of fracture zones

The serpentinization process (Fischer-Tropsch reaction) is a slow chemical reaction during which seawater transforms mantle peridotite into serpentine by hydration of olivine, ortho- and clinopyroxene. Methane and talc, which reduce the strength along faults^17^, are also formed during this reaction. Numerous examples of faults, carrying seawater across the brittle crust down to the upper mantle, exist in different tectonic environments. For example, during rifting seawater penetrates along active normal faults and serpentinizes the underlying mantle peridotite if the crust is entirely brittle^18,19^. In the Alps, peridotites are systematically serpentinized along mantle portions of faults^20^.

In the brittle oceanic domain, extension processes occur in the flexural bulge of subduction zones, as far as ∼100 km from the trench and give rise to a few km spaced parallel-to-the-trench normal faults reaching the upper mantle^5^. Observed low mantle velocities result from a combination of seawater-filled fractures, which serpentinize mantle peridotite^7^. We suggest that along the re-activated FZs of the Wharton basin seawater is conveyed during each significant tensional earthquake through sub-vertical sedimentary faults, through the brittle oceanic crust, and then down into the upper mantle, which is brittle down to a minimum depth of 30 km for a 55–60 m.y. old lithosphere^21^. The water access in mantle peridotites is controlled by the porosity and permeability of the medium^7^. The serpentinization process increases the volume of rocks and therefore reduces its porosity considerably. However, as the volume expansion also increases the permeability, the water flow might use these cracks to further develop the serpentinization process^18^. In consequence it can be assumed that the mantle part of sub-vertical re-activated FZs consists of serpentinized peridotite with lower seismic velocities than the surrounding pristine peridotite. As the damage zone in the mantle is sub-vertical and a few hundred meters wide, MCS profiles will not record such sub-vertical features. Therefore, even if FZs are not imaged in the crust and upper mantle, a horizontal seismic ray hitting perpendicularly a sub-vertical mantle damage zone with a low velocity anomaly will give rise to a reflection of 180° rotated polarity. However, Korenaga^22^ suggests that serpentinization might be less than assumed by all the authors cited above. These small crack-like porosities produced by thermal cracking and enhanced by bending-related faulting, do not necessarily lead to the substantial mantle hydration because of the high confining pressure. Therefore, mantle serpentinization would still occur but to a lesser extent^22^.

Within the oceanic crust, the damage zone consists of crushed rocks of brittle oceanic crust giving rise to mylonites, i.e. to a high density and high velocity medium. As for the mantle damage zones, such a narrow sub-vertical zone cannot be imaged by MCS data. On both sides of the damage zone, the nature of brittle oceanic crust is identical and therefore a horizontally travelling seismic ray crossing perpendicularly to the FZ will be reflected without changing polarity. Thus, only sedimentary deformation and associated sediment-buried basement fault scarps can help to approximately locate the position of the crustal and mantle portions of FZs.

### Upper mantle out-of-plane reflections

The computation of crustal and upper mantle out-of-plane reflections occurring perpendicularly to FZs is performed by using the RayInvr software^23^ and then projected along the plane of MCS Profile WG3 (in-plane reflections). In a first approximation, the geometry of oceanic crust and sedimentary layers only slightly varies along Profile WG3 (Fig. 3a). The two way travel time (TWTT) computation of ray paths from the current point D to the point projected perpendicularly to a FZ (E in Fig. 1b) only requires the use of a 1D velocity model of the oceanic lithosphere. This velocity model is extracted from refraction Profile R^24^ (Figs 1b and 2a), at the intersection with the eastward extension of Profile WG3 (see Methods section). The 1D velocity model is used to compute travel-times along DE (Fig. 1b). We assume that the ABC ray path is reflecting perpendicularly to the low velocity serpentinized damage zone acting as a mirror in the upper mantle (Fig. 2a). The trend of these out-of-plane reflections on FZs can be computed either in time or in space domains. As Profile WG3 and its interpretation, are displayed in time sections^4^ (Fig. 3a), computations are done in the time domain.

Out-of-plane reflections perpendicular to the FZ direction and the resulting projections on Profile WG3 are computed using the RayInvr software^23^, which is developed to process wide-angle ocean bottom seismometers data, and adapted here to the geometry of MCS data acquisition (See Methods section). We have not only explored the case of out-of-plane mantle reflections but also the case of out-of-plane crustal reflections on FZs (Figs 2c and 3, and See Methods section). Out-of-plane mantle reflections are recorded from 11.8 s downward and not in the upper mantle between 9.7 and 11.8 s (Fig. 3b and Supplementary Fig. 2a). Out-of-plane crustal reflections occur from 9.7 to 15.6 s, which gives a position from close to the Moho, in the lowermost part of the crust, down to ~38 km. Thus, the reflections identified in Fig. 3a between 9.7 and 11.8 s, i.e. within the uppermost ~7 km of mantle, correspond to out-of-plane crustal reflections. A significant overlap exists in the upper mantle between crustal and mantle reflection phases from 11.8 to 15.6 s, i.e. from depths of ~20 to ~38 km (Fig. 3, see Methods section and Supplementary Fig. 4b,c). In this depth range, it is impossible to distinguish between out-of-plane crustal and mantle reflections, except if a polarity change is observed for mantle reflections. Such a polarity change has been only mentioned for the deep mantle feature DMR1^4^ but may possibly exist for other observed deep events at distances of 45 and 195 km (Fig. 3a).

The location of re-activated FZs can be identified in gravity data (Fig. 1a) and in the sediments along Profile WG3 as F6a to F6d and F5^4,10^. Other re-activated FZs can be identified in swath-bathymetric data between F6a and F5^12,16^ and ~200 km southward^13^, and along the detailed sedimentary section of Profile WG3 (Supplementary Fig. 1a,c). How many re-activated FZs without gravimetric expression might exist between F5 and F6a? Are they able to produce artifacts that we have identified in Supplementary Fig. 2b and highlighted in Supplementary Fig. 1c? It is impossible to properly answer these questions. Though MCS data are unable to image sub-vertical features, we suggest that the upper mantle is largely fractured by numerous re-activated FZs.

When the seismic acquisition ship is close to a FZ (A’C in Fig. 2a), |BC| is null, and the distance from A’ to the FZ in the out-of-plane reflection is 9.5 km and 12.4 km along Profile WG3, respectively. The reflected paths resume symmetrically along Profile WG3, 12.4 km after the FZ crossing. The two symmetrical branches of mantle reflections with respect to a FZ are 24.8 km apart at a depth of 11.8 s (~22 km) (Fig. 3b). A variation of the in-plane reflection slopes along Profile WG3 (light pink area in Fig. 2b) based on ray-tracing of different mantle velocity gradients and extreme anisotropy values gives rise to variations of the in-plane reflection slopes of less than 1° (Supplementary Figs 2b and 3).

**Figure 4:**
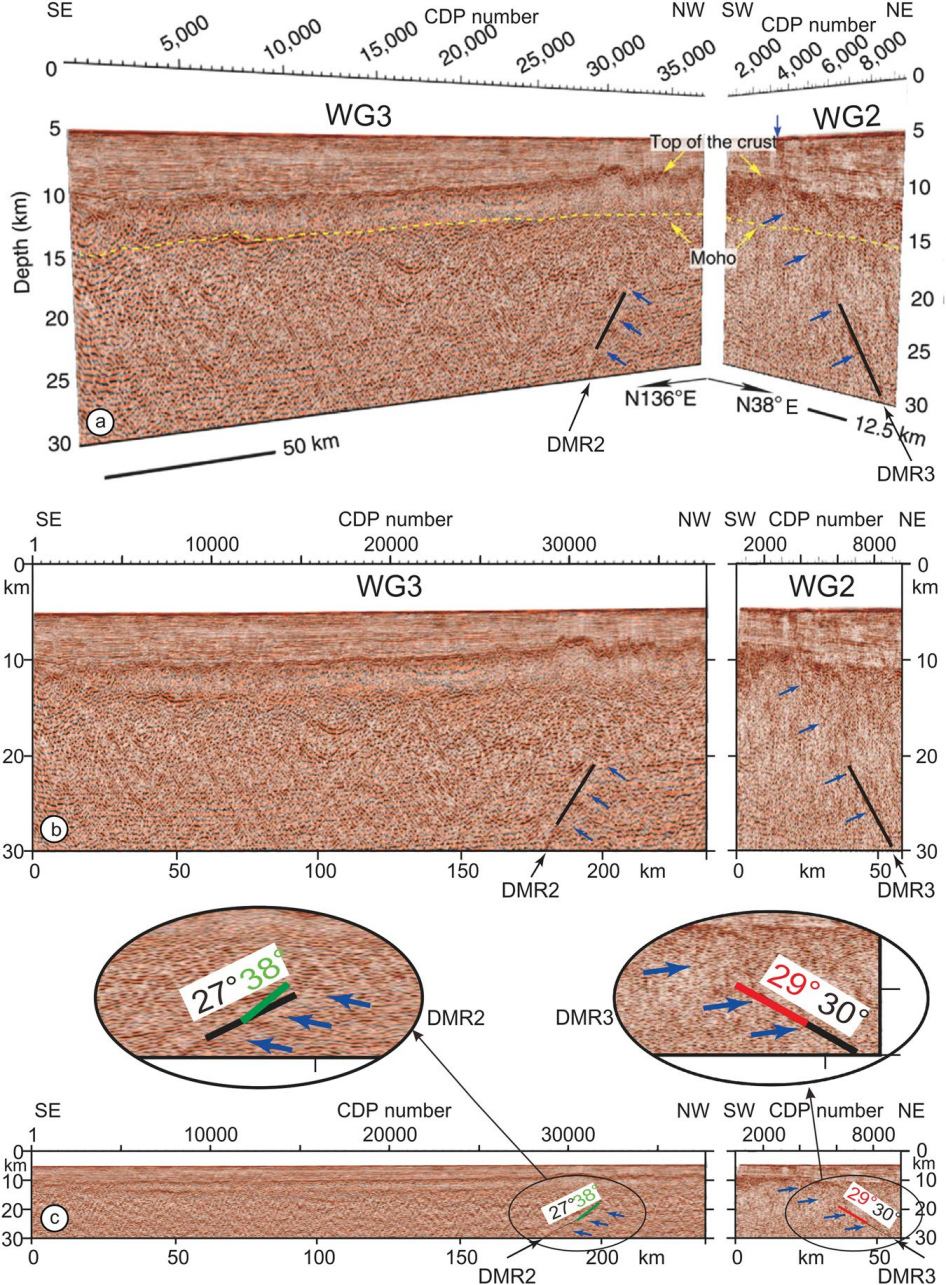
3D view of seismic sections along perpendicular profiles WG2 and WG3^4^ with a vertical exaggeration of ∼3. The two profiles (in depth domain) cut across F6b (Fig. 1b). Blue arrows underline deep mantle reflections DMR2 and DMR3 drawn by Qin and Singh (2015) and interpreted as faults by the authors. Solid black line: mantle portions of DMR2 and DMR3 where they are clearly defined. On Profile WG2, the Qin and Singh^4^ upward prolongation of the deep mantle reflection DRM3 to the sea-bottom (shown by their two upper blue arrows) is not convincing (see text). On Profile WG3, DRM2 does not extend within the crust. (**b**) 2D restoration of the two profiles. The upper mantle portions of DMR2 and DMR3 features (in black between depths of 20 and 30 km) correspond to clearly identified segments of features in (**a**). (**c**) Same profiles than in Fig. 2b without vertical exaggeration. In black, DMR2 and DMR3 mantle features with their dip values. Out-of-plane mantle reflections on F6b in the time domain were converted to the depth domain by using the two ways travel time (TWTT)/depth curve of Supplementary Fig. 5. Angles of Profiles WG3 and WG2 with F6b being 50° and 32° (Fig. 1b), the calculated dips of out-of-plane mantle reflections on F6b projected on Profiles WG2 and WG3 are 38° (green line) and 29° (red line), respectively. Depths of green and red segment extremities are calculated 1) for the minimum mantle reflection depth of 11.2 s extrapolated to the western extremity of Profile WG3 (Supplementary Fig. 4a), *i.e*. a depth of 19 km, and 2) for the computed TWTT of 12.6 s, which corresponds to the maximum depth of 25 km given by the 1D velocity model in Supplementary Fig. 4a. The coincident portion of the red line masks the upper part of DMR3 ending at a depth of 20 km.

The 1D velocity model was extracted from the refraction profile R, a few km east of Profile WG3 and applied to the complete profile WG3. However, the water depth, the thicknesses of sedimentary and crustal layers progressively change along Profile WG3: from east to west the water depth decreases (from 6.3 s to 5.8 s TWTT in Supplementary Fig. 1a), the sedimentary thickness reduces (from 2.7 s to 2.0 s in Supplementary Fig. 1a) and the crustal thickness increases (from 1.3 s to 1.6 s in Supplementary Fig. 1b). The 1D velocity model has been extrapolated to the end of Profile WG3 (blue curve in Supplementary Fig. 4a). Out-of-plane and in-plane crustal and mantle reflections are computed from the extrapolated 1D velocity model (Supplementary Fig. 4b) and compared to the 1D velocity model located east of Profile WG3 (Supplementary Fig. 4c). The first conclusion is that the slopes of the in-plane crustal and mantle reflections are almost identical (within a < 1° error) when using the blue or the red 1D velocity curves of Fig. 4a. The second conclusion is that crustal out-of-plane reflections along Profile WG3 are initiated in the extreme lowermost part of the crust, at a maximum distance of ~1 s above the Moho, both to the east and west of Profile WG3. Following Qin and Singh^4^ interpretations (Fig. 3), some mantle features extend about ~0.4 s in the lowermost crustal portion of Profile WG3.

In conclusion, the maximum expected variations of mantle velocities, the along-profile variations of water depths, sedimentary and crustal thicknesses, and the variations of vertical velocity curves only modify the slopes of the in-plane reflections along Profile WG3 by ~2°. In addition, the depth and range variations of out-of-plane crustal and mantle reflections do not vary more than 0.6 s TWTT, showing the robustness of the computations. Therefore, the out-of-plane mantle and crustal reflections along Profile WG3 can be directly compared (solid and dashed white lines, respectively) as displayed in Fig. 3a. The observed mantle features and computed out-of-plane reflected ray paths fit well even if it is impossible to attribute the out-of-plane reflections to a specific FZ due to the limited length of Profile WG3. For DMR1, the associated fault is probably located at a distance of 40 km in the sediments (Supplementary Fig. 1c), possibly associated with the basement scarp at 45 km. We do not see the southeastern symmetrical part of the ray path starting at a distance of 35 km and at a depth of 11.8 s. For DMR2, the associated fault might be F6b (Fig. 3a). The northwestern part of the ray path starts at a distance of 190 km and at a depth of 11.8 s, and the symmetrical ray path starts at a distance of 215 km and also at a depth of 11.8 s, that is too close from the end of Profile WG3 to detect it. For the other three deep mantle reflections (continuous white lines), the symmetrical ray paths are outside of Profile WG3.

### Independent confirmation of upper mantle out-of-plane reflections

The conclusion of Kent *et al*.^3^ study is that, based on a single MCS profile, it is impossible to undoubtedly demonstrate that a specific crustal feature is either real or a fake. Though this statement must also apply to mantle features, several specific points are already in favor of the presence of artifacts in the seismic section rather than the existence of faults along Profile WG3. Amongst them we can cite: (a) numerous mantle features omitted in the interpretation of Qin and Singh (2015) (Supplementary Fig. 1c), (b) a symmetry between mantle features trending N038° and -N038°, (c) no systematic offsets that are observed at the intersections of mantle features with opposite dips, (d) mantle features with almost no extension in the oceanic crust, and (e) no clear connection to oblique crustal features. Unfortunately, there is no coincident three-component OBS profile which would have provided more compelling information for distinguishing the near-vertical reflections from sideswipes as shown by Ohira *et al*.^25^ in the southeast of Shatsky Rise. To overcome this difficulty, Kent *et al*.^3^ suggest fulfilling a second condition, by acquiring either a 3D seismic survey or a grid of 2D profiles to map peculiar crustal features in 3D, a proposition that is in most cases supposed to be too costly. However, two perpendicular MCS profiles, Profiles WG2 and WG3^4^, can unequivocally help to prove if mantle reflections are real or not.

Both profiles WG2 and WG3 intersect with F6b (Fig. 4a). According to Qin and Singh^4^ the trenchward dipping reflection DMR3 can be traced along Profile WG2 from the seafloor down to a depth of 30 km. The authors also assert that DMR2 and DMR3 are independent faults and that their likely cause of formation is “bending-related stresses combined with stresses caused by diffuse deformation in the Wharton basin”^4^. Profiles WG2 and WG3 being displayed in the depth domain, the dips of out-of-plane mantle reflections perpendicular to F6d have been calculated in the depth domain by using the two-way travel time/depth curve of Supplementary Fig. 5. Errors in calculated dips are ~2° when taking into account the maximum expected variations of mantle velocities and along-profile variations of water depths, sedimentary and crustal thicknesses. The uncertainty due to the distance between the wide-angle seismic profile and the WG3 profile might be taken into account as an additional ~1° error. The error in the determination of observed dips (black lines) is difficult to assess because of multiple sources of errors: geometrical transformation from the perspective view (Fig. 4a) to profiles without vertical exaggeration (Fig. 4c), picking errors on DMR2 and DMR3 mantle features on the depth profiles (Fig. 4c) where features are difficult to identify with respect to the same feature on the time profile (Fig. 3a). The error on observed mantle features dips is estimated to a minimum of 5°. The combined error from observed mantle features and out-of-plane reflections on Profile WG2 calculated dips can be a minimum of 8°. The difference between observed mantle features dips (black lines) and calculated out-of-plane mantle reflections projected on MCS profiles (colored lines) are 7° and 1° for DMR2 and DMR3 features, respectively (Fig. 4c), which is smaller than the 8° error on observed and calculated dips. This observation shows that DMR2 and DMR3 image the same feature and are fakes (out-of-plane reflections), not existing faults. The second condition being fulfilled, we can assert that some, if not most of the mantle features identified as faults by Qin and Singh (2015) are fakes on Profiles WG2 and WG3.

DMR2 and DMR3 mantle features dip 31° and ∼30° respectively (Fig. 4c). If they belong to the same eastward dipping plane as suggested by Qin and Singh^4^, even if profiles WG2 and WG3 cut across F6b at different angles (32° and 50° respectively), this plane will be roughly parallel to F6b and located 50 km away of F6b at a depth of 30 km. It does not correspond to a FZ as it is too far to be sub-vertical and does not correspond to any expected crustal or mantle fault. However, if we assume that DMR2 and DMR3 features are out-of-plane reflections on F6b, there is no need to advocate a combination of stresses of different origins as suggested by Qin and Singh^4^. Consequently, DMR2 and DMR3 do not belong to a single eastward dipping fault or to two distinct dipping faults as suggested by Qin and Singh^4^. They are two artifacts, which correspond to two out-of-plane mantle reflections on F6b.

Ranero *et al*.^5^ and Grevemeyer *et al*.^26^ have shown swath-bathymetric and MCS examples of bending-related normal faults of incoming plates at the Middle America and Chile trenches creating a pervasive system of <5-km spaced faults that cut across the crust and penetrate deep into the mantle. Though Graindorge *et al*.^16^ interpret NW-SE trending seafloor structures as surface expression of bending-related normal faults near the Sumatra trench, in the area of Profile WG3, MCS lines presented by Geersen *et al*.^27^ show that the NW-SE trending structures observed in this area relate to underlying conjugate fault pairs interpreted as Riedel faults: the relative plate motions generate NW-SE oriented P axes, compatible with the main left-lateral re-activation of ~N-S trending FZs and the formation of conjugate Riedel shears only occurring within the sedimentary column and developed between FZs in response to transpression. The net consequence of these observations is that bending-related stresses do not create oceanic faults in the Wharton basin. Only re-activated FZs located southwest of northern Sumatra break the brittle oceanic crust and upper mantle before being subducted.

### Questions raised by mantle features

Why do the mantle features display a symmetry with respect to a vertical plane? The dip of mantle reflections varies between 25° to 35°^4,10^. However, these authors do not clearly mention the symmetry between the two families of features, even if this symmetry is readily identifiable. In our hypothesis, the detailed image of WG3 in the sediments^4,10^ (Supplementary Fig. 1) suggests that between F6 and F5, several sub-vertical faults with vertical offsets can be identified as re-activated FZs, which extend at depth within the crust and upper mantle. We thus suggest that most of the mantle features dipping in both directions are out-of-plane reflections on the crustal or mantle parts of the FZs, which give rise to symmetrical dipping artifacts when crossing the FZs.

Why can no systematic offset be observed at the intersections of mantle features of opposite dips? In general, when two families of conjugate faults intersect, one of them is offset. This is not observed at the intersections of most of mantle features (Fig. 3a and Supplementary Fig. 1b,c). This point is not discussed in Qin and Singh^4^ and Carton *et al*.^10^. Following our work, there is no reason to see offsets at the intersections of mantle features of opposite dips, as these features are simply artifacts.

Why do mantle features reach up close to the Moho interface with almost no extension into the oceanic crust and no connection to oblique crustal features? Even with high quality MCS data, Qin and Singh^4^ do not provide images showing a continuation of mantle faults within the oceanic crust and overlying sediments, except for DRM3, which is not an indisputable example, at least on their figure (Fig. 4). All mantle features terminate near the Moho. In our model, in-plane crustal reflections appear in the upper mantle, and possibly, due to the use of a 1D velocity model, in the extreme lower part of the crust (Fig. 3). Note the overlap between the crustal and mantle reflections in the uppermost mantle.

Why does the polarity of seismic signal change for the deep mantle feature DMR1? Qin and Singh^4^ suggest that the negative polarity along DRM1 might be due to seawater penetrating along crustal fault damage zones down to the upper mantle. The mechanism of seawater transfer is associated with the presence of thrust faults active several tens of m.y. after the oceanic crust formed in order to disrupt the brittle upper mantle, or is associated with the presence of large earthquakes re-activating N335° normal faults of the original oceanic fabric. Though Qin and Singh^4^ only mention the negative polarity along DMR1, in our model, we suggest the presence of narrow, sub-vertical low velocity zones along the mantle part of all re-activated FZs as a consequence of the more or less developed serpentinization process. The degree of serpentinization being highly variable along the FZs^7,18,19,22^, the reflection point on the mantle serpentinized portion of the mantle moving several tens of km (Fig. 1c,d), the discontinuity of observed features may be explained by laterally and/or vertically inhomogeneous serpentinization processes or by localized morphological heterogeneities along the sub-vertical damage zones.

Even if answers to previous questions are in favor of the presence of numerous fakes rather than faults in the upper mantle, the two perpendicular profiles WG2 and WG3 cut across the same FZ and display dipping features down to a depth of 30 km. These two features can be merely explained as out-of-plane reflections on this FZ. Thus, even if MCS profiles do not image sub-vertical FZs, preventing the identification of conjugate features, some if not all mantle features observed along Profiles WG3 and WG2 are fakes rather than faults. More generally, oblique oceanic mantle features^28,29^ should alert scientists to be cautious in their interpretation of oceanic mantle features. These calculations should also be taken into account when planning 2D reflection seismic surveys.

## Methods

### Velocity model

The vertical velocity model used for computations (Fig. 2a) is derived from refraction velocities obtained from the southern part of Profile R shot perpendicularly to Profile WG3^24^ (Fig. 1b). Five sedimentary layers with velocity gradients were identified. Crustal velocity and gradient are typical of oceanic crust^30^. Based on tomographic inversion data, the mantle velocity is ∼8.0 km/s^24^. Depending on profile orientations with respect to the spreading direction, upper mantle velocities may display 7–10% anisotropy, with Pn velocities varying from 7.9 to 8.7 km/s in the northwest Pacific basin on profiles perpendicular or parallel to the spreading direction^31^, respectively. In the Wharton Basin, we assume that Vm might vary from 8.0 to 8.7 km/s for profiles perpendicular to the FZ direction (Fig. 2).

### RayInvr software

**Figure 5:**
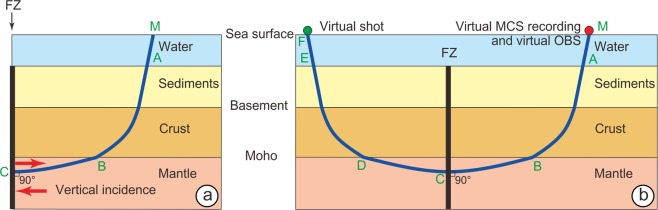
Schematic diagram of out-of-plane reflection ray paths. The MCS reflections are calculated by using the RayInvr software^23^ developed to process wide-angle seismic data. (**a**) If we assume that the seismic ray is reflected perpendicularly to the FZ acting as a mirror for the out-of-plane mantle reflections, the ray path is MABCBAM. (**b**) If D, E and F are symmetrical to B, A, and M with respect to the vertical plane of the FZ, MABCBAM = MABCDEF. Then, ray paths travel times and offsets are computed by using the RayInvr software.

**Figure 6:**
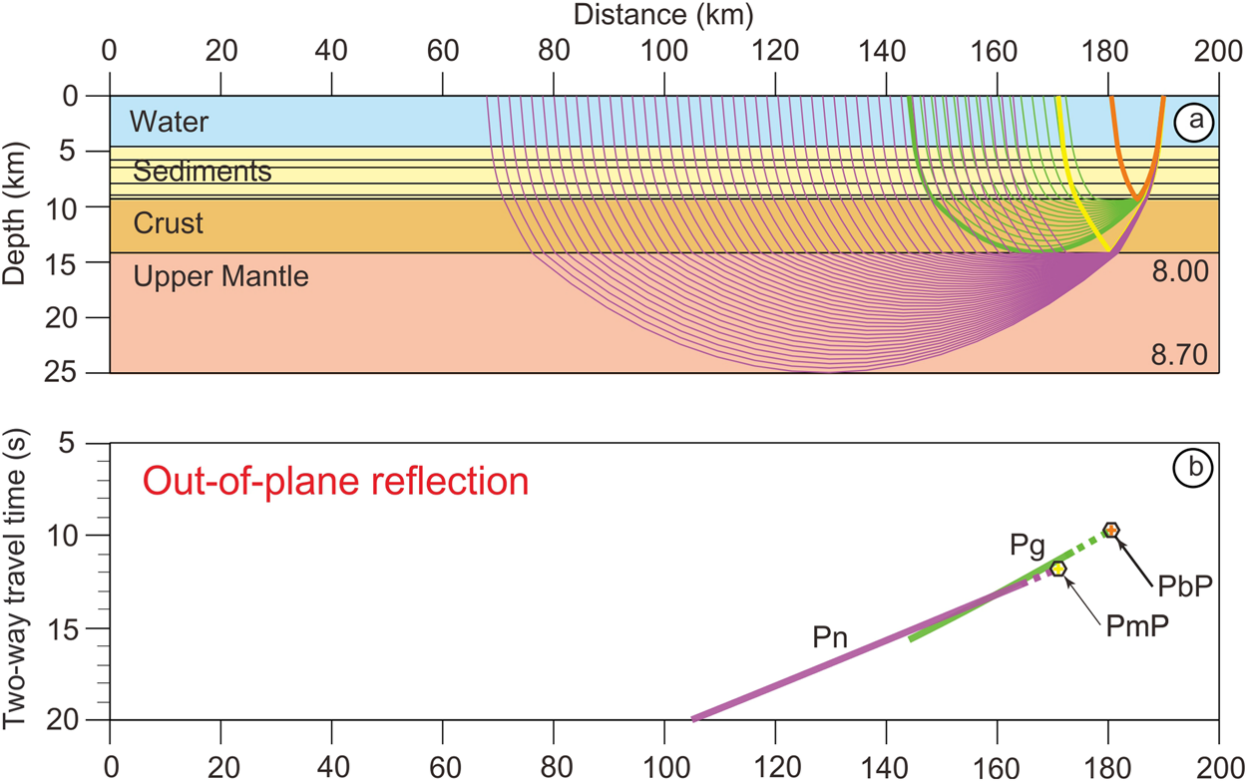
Ray tracing and travel times simulations for different ray paths. (**a**) Ray paths in the 2D velocity model of Fig. 2a. (**b**) Calculated travel times. Ray paths in different colors in (**a**) correspond to travel times of similar colors in (**b**). PbP is the ray path reflected on the top of the crust (thick orange continuous line in (**a**) and orange polygon in (**b**)). PmP is the reflection from the Moho (thick yellow continuous line in (**a**) and yellow polygon in (**b**)). Pg is the crustal reflective phase (in green) starting from the orange polygon down to 15.66 s (Supplementary Fig. 4c) at 144 km, distance at which the Pg refraction phase is tangent to the base of the crust. Pn is the phase turning in the upper mantle (in purple) starting from the yellow polygon down to 20 s.

If we assume that the seismic ray is reflected perpendicularly to the FZ acting as a mirror for the out-of-plane mantle reflections, the ray path is MABCBAM (Fig. 5a). If D, E and F are symmetrical to B, A, and M with respect to the vertical plane of the FZ, MABCBAM = MABCDEF. Ray tracing, travel times and offsets are computed by using the RayInvr software^23^ (Fig. 6) and are appropriate for a comparison with migrated MCS sections either in time or in space domains as reflectors are replaced in their correct positions.

## Supplementary information

Supplementary Information
