# Supplementary material for: Oceanic mantle reflections in deep seismic profiles offshore Sumatra are faults or fakes
Source: Sci Rep. 2019 Sep 16;9:13354. doi: 10.1038/s41598-019-49607-x (PMC6746736; doi:10.1038/s41598-019-49607-x)
Supplement: Supplementary file 1 — Supplementary Information [file 41598_2019_49607_MOESM1_ESM.docx]

**Oceanic mantle reflections in deep seismic profiles offshore Sumatra are faults or fakes**

Jean-Claude Sibuet^1, 2, 3^, Enyuan He^1, 4^, Minghui Zhao^1, 4, 5 *^, Xinming Pang^1, 5^ and Frauke Klingelhoefer^3^

1. Key Laboratory of Ocean and Marginal Sea Geology, South China Sea Institute of Oceanology, Chinese Academy of Sciences, Guangzhou 510301, China
2. 44 rue du Cloitre, 29280 Plouzané, France
3. Ifremer Centre de Brest, B.P. 70, 29280 Plouzané Cedex, France
4. Innovation Academy of South China Sea Ecology and Environmental Engineering, Chinese Academy of Sciences, Guangzhou 510301, China
5. University of Chinese Academy of Sciences, Beijing 100049, China

Corresponding author: Minghui Zhao ([mhzhao@scsio.ac.cn](mailto:mhzhao@scsio.ac.cn))


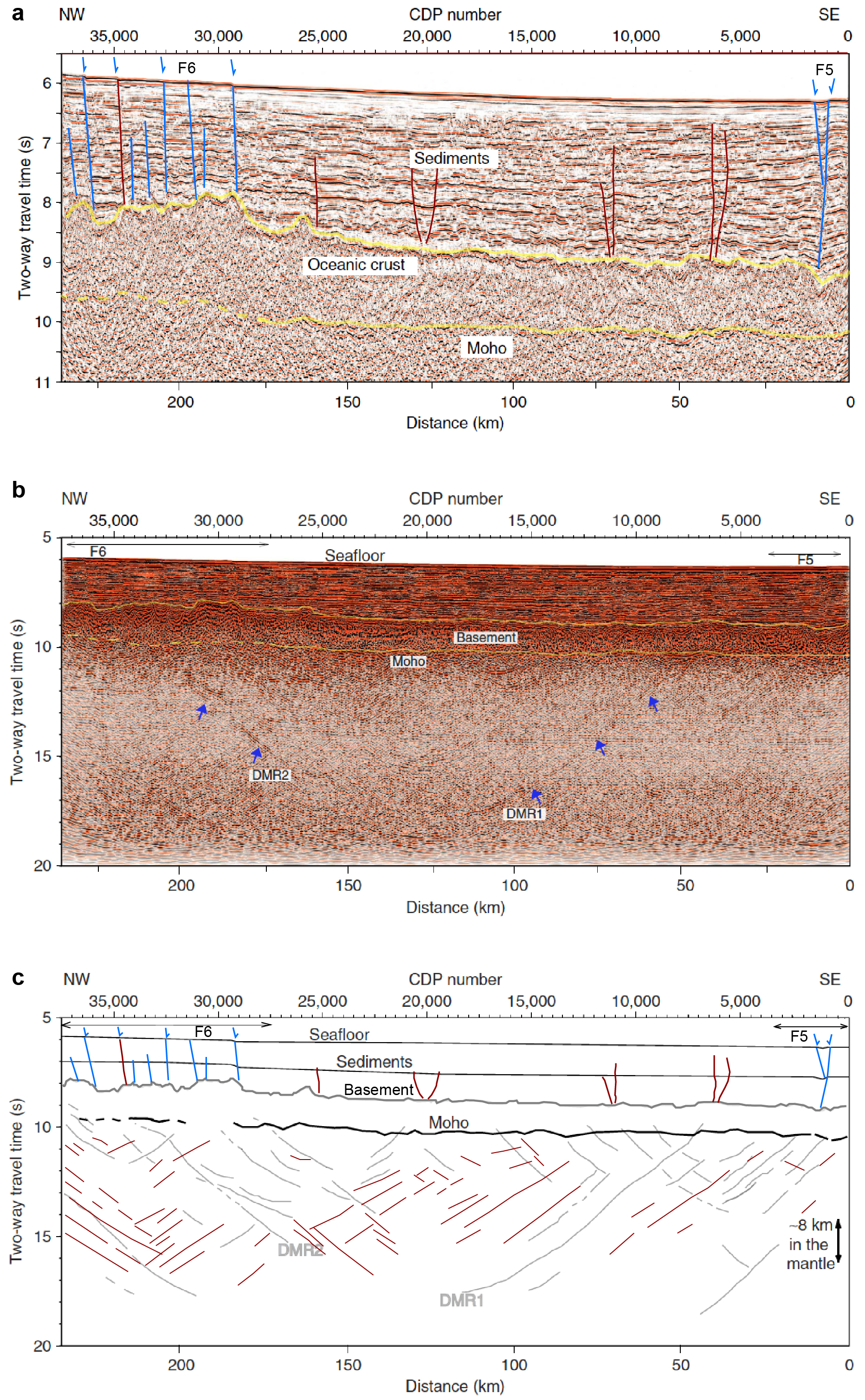


**Supplementary Figure 1. Images of MCS Profile WG3.** (**a**) Shallow part of WG3 image in time domain with faults in the regions of FZs F5 and F6 marked by blue lines and arrows (modified from Qin and Singh^4^). Some of these FZs, imaged ~200 km southward on swath-bathymetric data show that they are left-lateral re-activated FZs^13^. Between F5 and F6, several re-activated FZs (dark purple lines) are imaged. (**b**) WG3 post-stack time domain migrated seismic reflection image down to 20 s TWTT. **(c)** Line drawing of interpreted mantle reflections as faults (light grey lines) ^4^, with DMR1 and DMR2 the deepest mantle faults. A detailed inspection of (b) suggests that numerous mantle reflections have been omitted (dark purples lines). In general, these features dip northwest in the southeastern portion of the profile and southwest in the northwestern portion of the profile. Most of them, including those identified by Qin and Singh^4^, may be artifacts because their trends are suspiciously parallel.


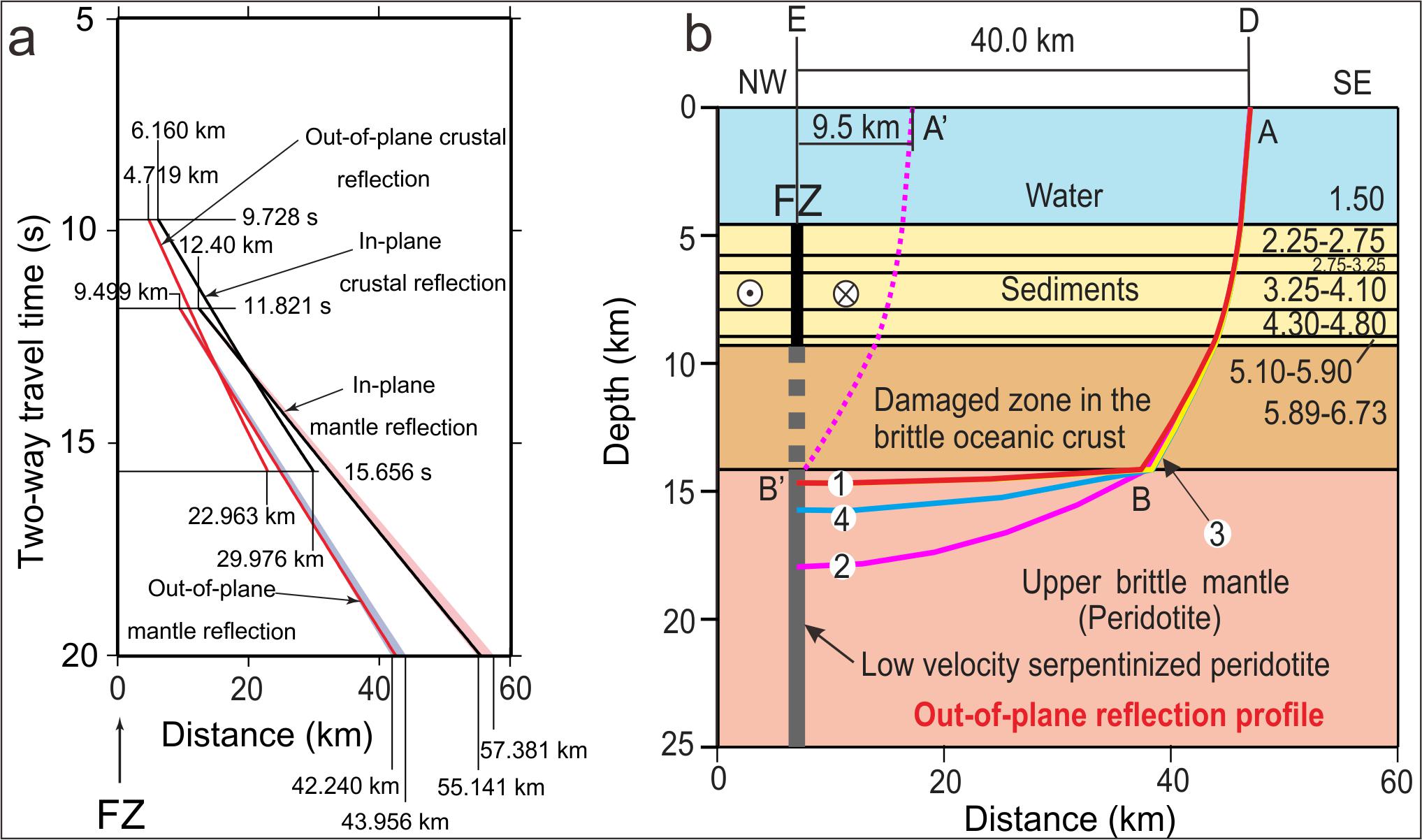


**Supplementary Figure 2. Out-of-plane crustal and mantle reflections projected on WG3 Profile (in-plane reflections) and ray tracing for four velocity models. a)** Computations are performed using the 1D velocity profile extracted from the wide-angle profile R (Fig. 1b) ^24^, 24 km at its southern end. Mantle velocities are varying from the Moho to a depth of 25 km from 8.0 to 8.1 km/s, 8.0 to 8.7 km/s, 8.4 to 8.5 km/s and 8.4 to 8.7 km/s. The light blue and light red areas include extreme solutions of above mantle velocity distributions. A significant overlap exists between crustal and mantle reflections. **b)** To take into account mantle velocity variations due to anisotropy (see Methods), from the Moho to a depth of 25 km, mantle velocities vary from 8.0 to 8.1 km/s (model 1, red), from 8.0 to 8.7 km/s (model 2, pink), from 8.4 to 8.5 km/s (model 3, yellow), and from 8.4 to 8.7 km/s (model 4, blue).


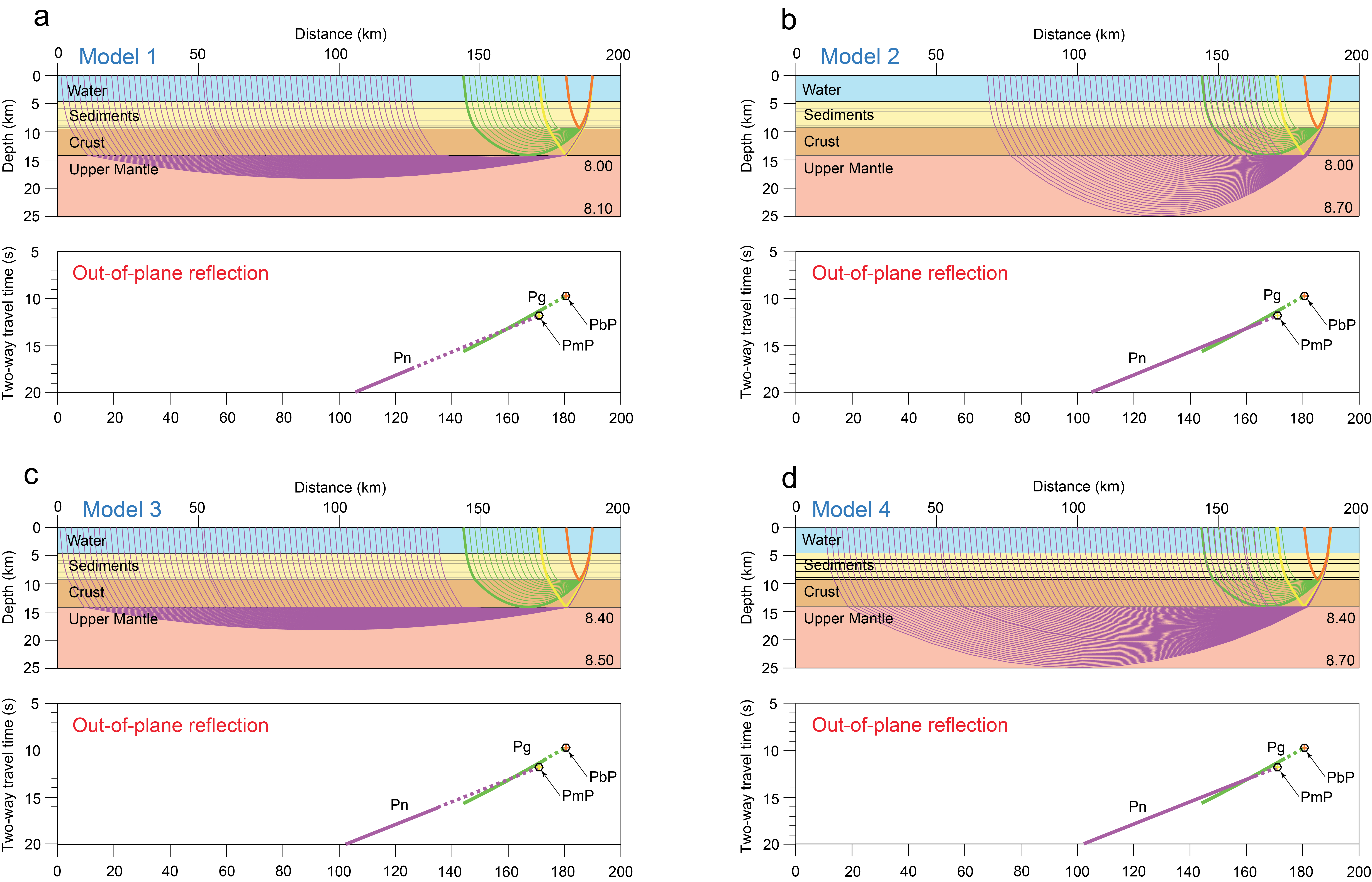


**Supplementary Figure 3: Calculations of out-of-plane reflections with RayInvr software.** Mantle velocities vary from the Moho to a depth of 25 km from 8.0 to 8.1 km/s (**a**), from 8.0 to 8.7 km/s (**b**), from 8.4 to 8.5 km/s (**c**), and from 8.4 to 8.7 km/s (**d**).


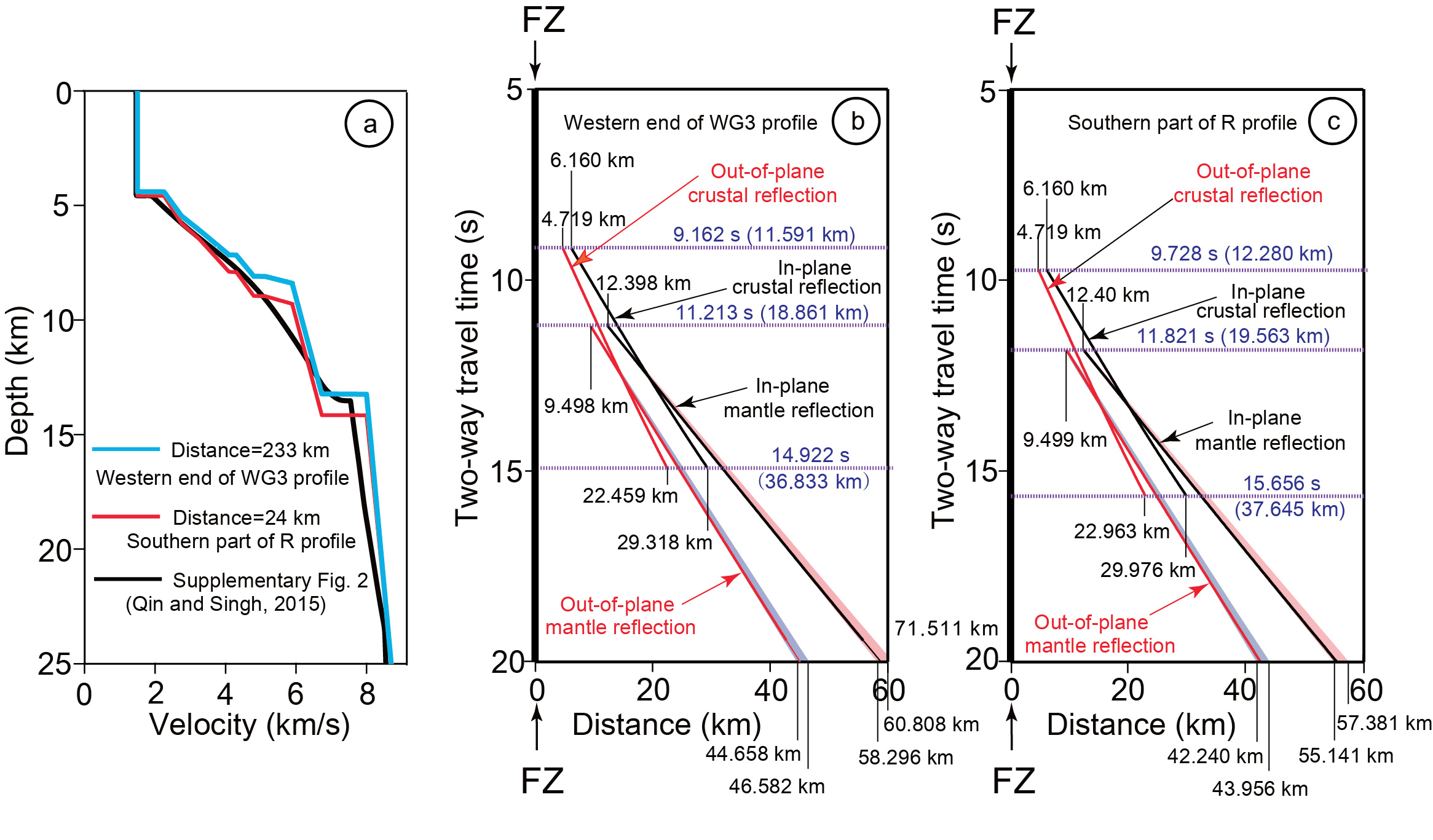


**Supplementary Figure 4. Extrapolation of 1D refraction velocity model to the western end of Profile WG3.** (**a**) 1D velocity profiles: In red, velocity profile extracted from the R profile, in the eastern prolongation of Profile WG3; In blue, previous profile extrapolated to the western extremity of Profile WG3. Compared to the eastern part of Profile WG3, west of Profile WG3 the water depth is smaller (5.8 s instead of 6.3 s TWTT in Supplementary Fig. 1a), the sedimentary thickness is smaller (2.0 s instead of 2.7 s in Supplementary Fig. 1a) and the crustal thickness is larger (1.6 s instead of 1.3 s in Supplementary Fig. 1b); In black, tomographic 1D velocity model of Qin and Singh^4^ along Profile WG2. Though smoother, this velocity profile (black curve) is located in between the two blue and red curves, except between depths of 9 to 12 km. (**b**) Out-of-plane and projected on WG3 Profile (in-plane) crustal and mantle reflections extrapolated from 1D refraction profile R^24^, 24 km from its southern end, to the western end of Profile WG3. Mantle velocities are varying from the Moho to a depth of 25 km from 8.0 to 8.1 km/s, 8.0 to 8.7 km/s, 8.4 to 8.5 km/s and 8.4 to 8.7 km/s. The light blue and light red areas include extreme solutions of above mantle velocity distributions. A significant overlap of 3.8 s (18 km) exists between crustal and mantle reflected phases. Maximum and minimum depths of crustal reflections are given in seconds converted in km by using the blue velocity curve of Supplementary Fig. 4a. (**c**) Out-of-plane and projected on WG3 Profile (in-plane) crustal and mantle reflections extrapolated from 1D refraction profile R, 24 km from its southern end. Same legend than in (b). Maximum and minimum depths of crustal reflections are given in s converted in km by using the red velocity curve of Supplementary Fig. 4a.


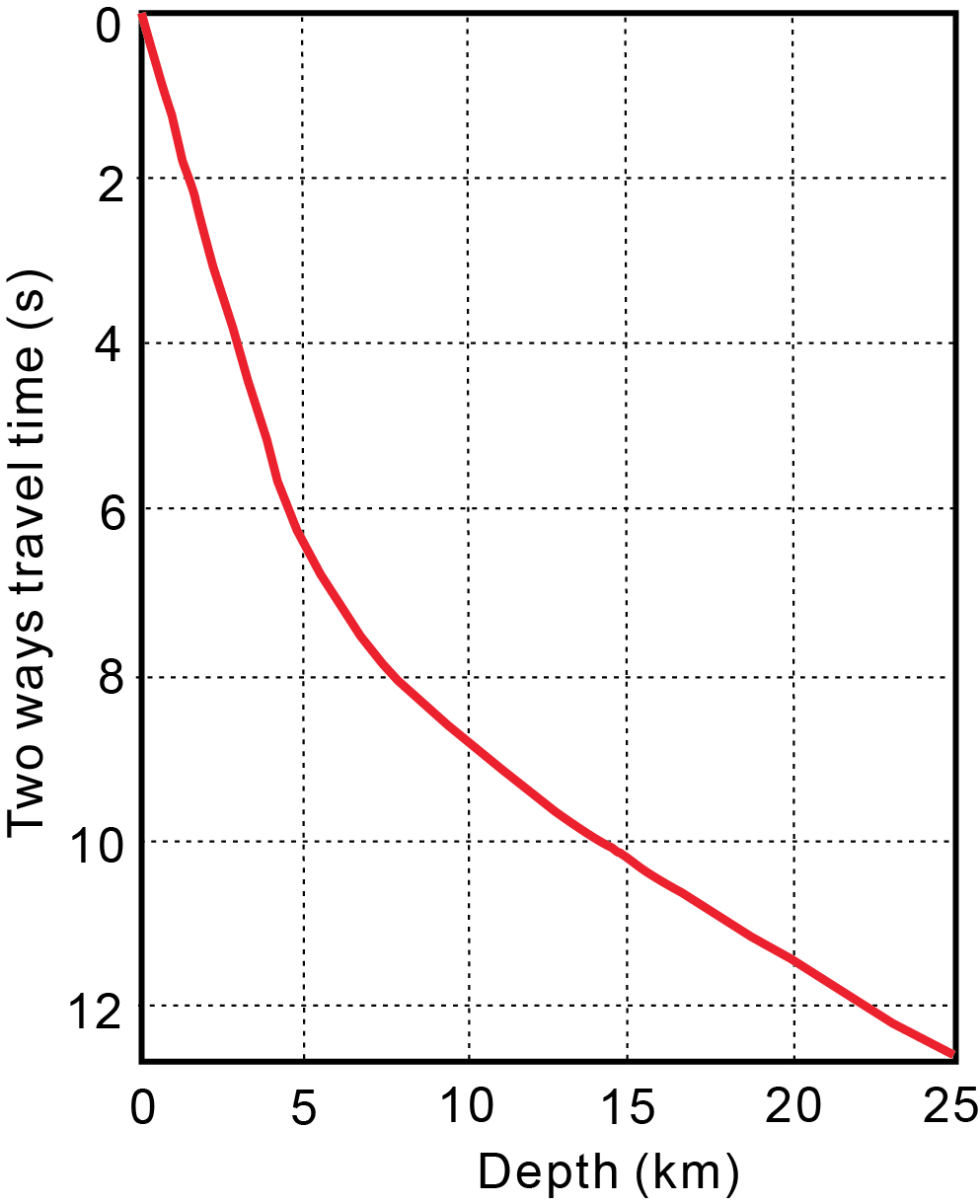


**Supplementary Figure 5. Two ways travel time/depth curve.** The two ways travel times (TWWT) are calculated at different depth points using the 1D model of oceanic crust from Profile R (Fig. 2a) and assuming that every depth point is a reflecting interface. At a depth of 25 km, TWTT is 12.6 s.
